# Hybrid neural network based on novel audio feature for vehicle type identification

**DOI:** 10.1038/s41598-021-87399-1

**Published:** 2021-04-07

**Authors:** Haoze Chen, Zhijie Zhang

**Affiliations:** 1 School of Instrument and Electronics, North University of China, Taiyuan, 030051 China; 2 Key Laboratory of Instrumentation Science and Dynamic Measurement (North University of China), Ministry of Education, Taiyuan, 030051 China

**Keywords:** Electrical and electronic engineering, Mechanical engineering

## Abstract

Due to the audio information of different types of vehicle models are distinct, the vehicle information can be identified by the audio signal of vehicle accurately. In real life, in order to determine the type of vehicle, we do not need to obtain the visual information of vehicles and just need to obtain the audio information. In this paper, we extract and stitching different features from different aspects: Mel frequency cepstrum coefficients in perceptual characteristics, pitch class profile in psychoacoustic characteristics and short-term energy in acoustic characteristics. In addition, we improve the neural networks classifier by fusing the LSTM unit into the convolutional neural networks. At last, we put the novel feature to the hybrid neural networks to recognize different vehicles. The results suggest the novel feature we proposed in this paper can increase the recognition rate by 7%; destroying the training data randomly by superimposing different kinds of noise can improve the anti-noise ability in our identification system; and LSTM has great advantages in modeling time series, adding LSTM to the networks can improve the recognition rate of 3.39%.

## Introduction

In modern transportation system, the application of classifying different vehicle types is quite crucial in daily life, such as intelligent monitoring system, auto-charging system and illegal preemption of way detection. Machine learning has been more and more widely used in image recognition^1^, speech recognition^2^, natural language processing^3^ and other fields^4–6^. Machine learning can be divided into^7^, unsupervised learning^8^, semi-supervised learning^9^, and enhanced learning^10^. Supervised learning means a learning method that assigns labels or labels to training data. Supervised learning mainly deals with classification and regression problems. Unsupervised learning does not give any label or label answers to the training data. It often performs cluster analysis classification and outlier detection on these data. Semi-supervised learning, as the name implies, is a combination of supervised learning and unsupervised learning. A part of the training data of the machine has tags or answers, and the other part does not. Reinforcement learning is also called reinforcement learning. It takes actions according to the surrounding environment, learns and adjusts the action based on the results and feedback of each action. It must learn what is the best strategy to get the most rewards over time. A growing number of scholars are adopting machine learning approach to classify vehicles by training a large number of images. Wang et al. proposed an approach to detect vehicles from aerial images with different resolutions and perspectives in an approximate real-time manner^11^. Huang et al. propose a simple and effective vehicle detection method based on local vehicle's texture and appearance histograms feed into clustering forests^12^. However, it is not possible to capture vehicle images clearly due to complex lighting and harsh weather. The effect of vehicle identification by acquiring the images is not satisfied.

However, collecting audio is not affected by harsh weather, such as fog and heavy rain. The identification system using vehicle audio is very robust. And it is more convenient than the image collection. Yuan studied a novel detection model to give attention both the recognition accuracy and the detection efficiency and so on in the existing vehicle identification and classification^13^. Inagaki k showed that using recurrent neural network can classify vehicle type accurately^14^. Fan of Tsinghua University studied composed wavelet transform and mutual information to recognize vehicle^15^. The vehicle type identification based on audio signal divides into two processes: the feature extraction and the identification process. Extracting feature is the process of extracting comprehensive information from vehicle audio signals; after extracting, a suitable classifier is designed to identify the vehicle type.

Here, we extract three kinds of features including the Mel frequency cepstrum coefficients, the pitch class profile and the short-term energy and splice them as the final feature.

## Extraction and splice of vehicle features

Acoustic characteristics, perceptual characteristics and psychoacoustic characteristics make up audio characteristics. Time and frequency domain parameters constitute acoustic characteristics, including the short-time energy, the short-time autocorrelation function, and the short-time zero crossing rate and so on. Perceptual characteristics are the parameters extracted from human auditory, including the Mel Frequency Cepstrum Coefficients, and its first-order and second-order differential which are used to reflect the dynamic characteristics. Psychoacoustic characteristics include loudness, roughness and pitch class profile etc.

### Mel frequency cepstrum coefficient (MFCC)

**Figure 1:**
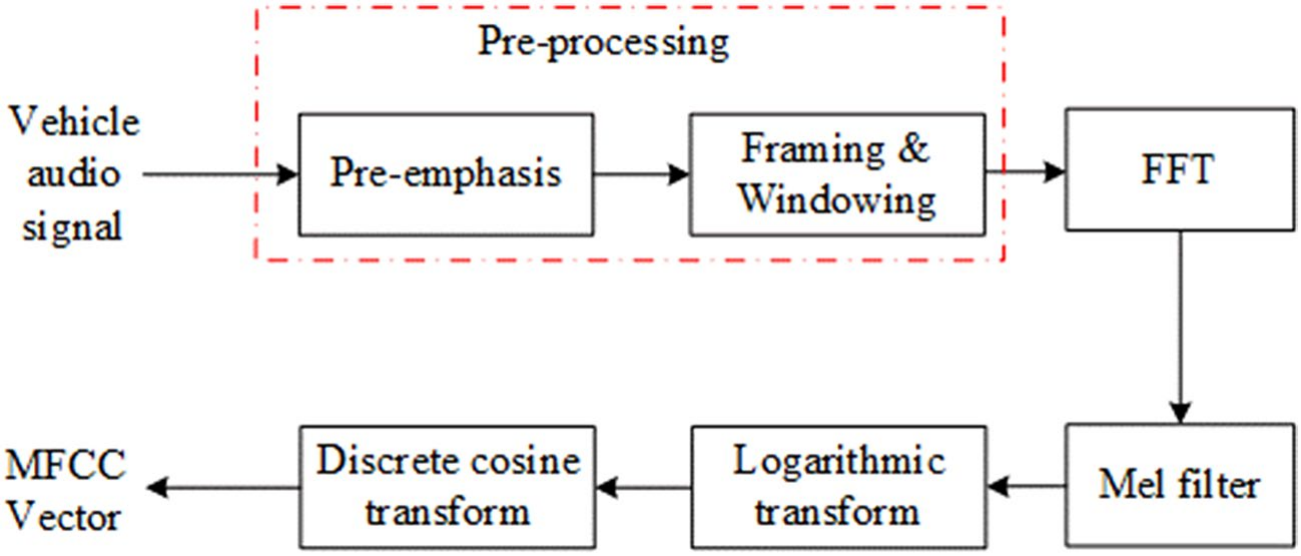
The process of extracting MFCC.

The Mel frequency cepstrum coefficient was first used in the field of automatic speech recognition (ASR) and speaker recognition. MFCC is a kind of cepstral coefficient based on Mel frequency. After the pre-processing of vehicle audio signals, MFCC vector will be extracted from each frame in the form of vector group. Fast Fourier Transform (FFT) is applied to each frame of the audio signals before sending to Mel filter where the original spectrum is transformed into Mel frequency spectrum. Then logarithmic transformation and discrete cosine transform are applied to the spectrum to form MFCC vectors. Equations (1) to (4) reflect the entire transformation process. Figure 1. shows the process of extracting MFCC.

1 $$ s(k) = \sum\limits_{m = 0}^{N - 1} x (m)e^{( - j2\pi mk/N)} ,0 \le k \le N $$

*x*(*m*) is the input audio signal, *N* is the number of Fourier transform.

Then we use M Mel filter banks to filter the audio signal. The transfer function of the *m*th filter bank can be expressed as follows:

2 $$ H_{m} (k) = \left\{ {\begin{array}{*{20}c} 0 & {k < f(m - 1)} & {} \\ {\frac{2(k - f(m - 1))}{{(f(m + 1) - f(m - 1))(f(m) - f(m - 1))}}} & {f(m - 1) \le k \le f(m)} & {} \\ {\frac{2(f(m + 1) - k)}{{(f(m + 1) - f(m - 1))(f(m + 1) - f(m))}}} & {f(m) \le k \le f(m + 1)} & {} \\ 0 & {k > f(m + 1)} & {} \\ \end{array} } \right. $$

where, $\sum\limits_{m}^{M - 1} {H_{m} } (k) = 1$ , $f\left( m \right)$ is the center frequency of the triangular filter. Then the logarithmic transformation of the *m*th filter bank can be expressed as:

3 $$ s(m) = \ln \left( {\sum\limits_{k = 0}^{N - 1} | s(k)|^{2} H_{m} (k)} \right)\quad 0 \le m \le M $$

Finally, we perform a discrete Fourier transform on a to obtain the Mel frequency cepstrum coefficient:

4 $$ C(n) = \sum\limits_{n = 0}^{M - 1} s (m)\cos (\pi n(m + 0.5)/M)\quad 0 \le n \le M $$

where, M is the dimension of the MFCC feature.

### Pitch class profile (PCP)

**Figure 2:**
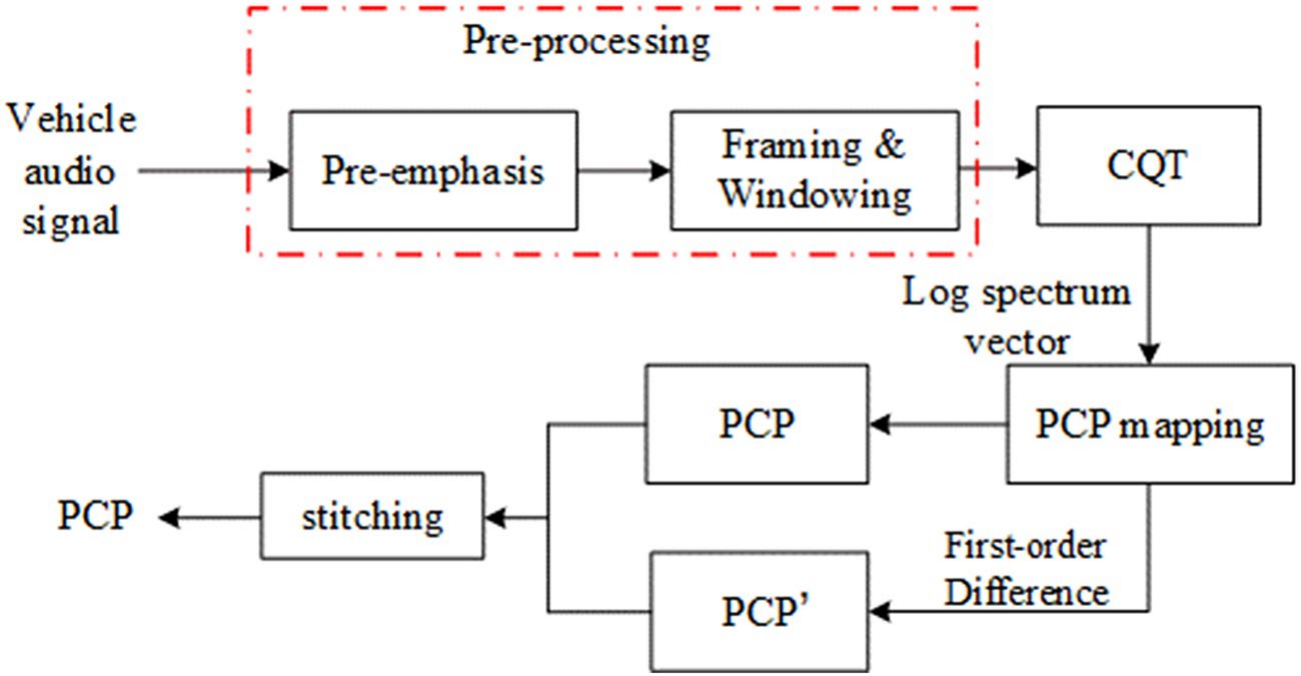
The process of extracting PCP.

The pitch class profile can be used to extract the characteristics of the audio tone. It reconstructs the spectrum into sound spectrum, which describing the level of sound^16^. When extracting the MFCC, we send the audio signals to Mel filter to smooth spectrum and eliminate harmonics. The formant of audio will be highlighted. So the tone characteristics of vehicle audio will be ignored. But the vehicle audio contains complex audio signals including horn sound, tire friction sound and engine sound, etc. So the pitch class profile can be selected to be the characteristic to recognize different types of the vehicles. The process of extracting PCP is shown in Fig. 2.

The specific extraction steps are as follows:

Step 1: $x\left( m \right)$ is the collected audio signal, we segment the pre-processed audio into frames, N is frame length; *N/2* is frame shifting; we use Hamming window as window function,

5 $$ X^{cqt} \left( k \right) = \frac{1}{N}\sum\limits_{m = 0}^{{N_{k} - 1}} {x\left( m \right)w_{{N_{k} }} \left( m \right)e^{{ - j\frac{2\pi Q}{{N_{k} }}m}} } $$

The formula represents the spectrum of the *k*th semitone of the *n*th frame. $w_{{N_{k} }} \left( m \right)$ is the window and $N_{k}$ is the length of the window. *Q* is the constant factor of Constant-*Q* spectral analysis.

Step 2: Spectrum mapping. We map the spectrum $X^{cqt} \left( k \right)$ to *p*(*k*) in the pitch class domain, A 12-bin tuned Chromagram is then calculated from the Constant-Q spectra. The CQT spectrum is mapped to a pitch contour feature in a logarithmic manner according to the averaging law in music theory. *k* in $X^{cqt} \left( k \right)$ is mapped to *p* in *PCP*. The mapping formula is:

6 $$ p(k) = \left[ {12 \times \ln (k \cdot f_{s} {/}N \cdot f_{{r{\text{ef}}}} )} \right]\bmod 12 $$

In the formula, $f_{s}$ is sampling rate; $f_{s} /N$ is the frequency domain interval; $f_{ref}$ is the reference frequency, corresponding to the C1 in music; indicates the frequency of each frequency domain.

Step 3: Accumulate all the frequency which corresponding to the pitch class, we will obtain the 12-bin PCP vector:

7 $$ PCP\left( p \right) = \sum\limits_{k:p\left( k \right) = p} {\left| {X^{cqt} \left( k \right)} \right|} ,p = 1,2,...,12 $$

Step 4: In order to make the *PCP* more dynamic, we perform a first-order difference on the $PCP\left( p \right)$ to get $PCP^{\prime}\left( p \right)$ and then stitch $PCP\left( p \right)$ and $PCP^{\prime}\left( p \right)$.

### Short-term energy

The energy can reflect vehicle audio feature more comprehensively. $x_{n} \left( m \right)$ is the *n*th frame of pre-processed audio signal. $E_{n}$ is the short-term energy:

8 $$ E_{n} = \sum\nolimits_{m = 1}^{L} {\left[ {x_{n} \left( m \right)} \right]^{2} } $$

In the formula, *L* is the length of the frame.

We extract 40 dimensional MFCC vector ${\mathbf{T}}_{1} = [a_{1} ,a_{2} ,...,a_{40} ]$, 24 dimensional PCP vector ${\mathbf{T}}_{{2}} = [b_{1} ,b_{2} ,...,b_{24} ]$, and 1 dimensional short-term energy vector ${\mathbf{T}}_{{3}} = [{\text{c}}_{1} ]$, and stitch from beginning to end, obtaining 65 dimensional new. Feature ${\mathbf{T}}_{1} = [a_{1} ,...,a_{40} ,b_{1} ,...b_{24} ,c_{1} ]$.

## Hybrid neural network

### Convolutional neural network (CNN)

Essentially, CNN is an extended structure of multi-layer perceptron, the construction of the network layer structure has a great impact on the actual output. The classic convolutional neural networks include input layer, convolutional and pooling layer combined in various forms, a limited number of fully connected layer, and output layer. In the convolutional layer, the convolution kernel performs a convolution operation on the vector which export from the previous layer. Nonlinear system can be obtained by activation function, mathematical model can be expressed as:

9 $$ x_{j}^{l} = f(\sum\nolimits_{{i \in {\mathbf{M}}_{j} }} {x_{i}^{l} } \times k_{ij}^{l} + b_{j}^{l} ) $$

In the formula, ${\text{M}}_{j}$ is input vector; *l* is the layer *l* network; *k* is the convolution kernel; *b* is bias; $x_{j}^{l}$ is the output of layer *l*. We select ReLU as the activation function.

### Long short-term memory (LSTM)

**Figure 3:**
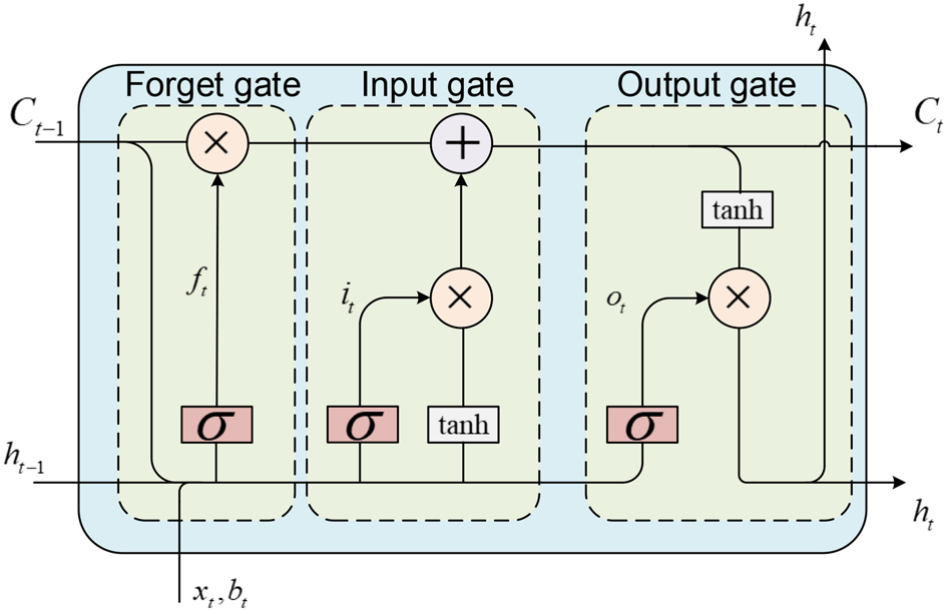
Structure of the LSTM unit.

Long short-term memory (LSTM) is a special kind of RNN^17^, mainly to solve the problem of gradient disappearance and gradient explosion during long sequence training. To put it simply, LSTM can perform better in longer sequences than ordinary RNN. A LSTM model includes the input gate *i*_*t*_, the output gate *o*_*t*_, the forget gate *f*_*t*_ and a cell *c*_*t*_. We can see the LSTM structure from Fig. 3.

The forget gate can be express as:

10 $$ f_{t} = \sigma \left( {W_{f} \left[ {x_{t} ,h_{t - 1} ,C_{t - 1} } \right] + b_{t} } \right) * C_{t - 1} $$

where, *x*_*t*_ is input unit; *h*_*(t−1)*_ is the output unit; *C*_*(t−1)*_ is the status of previous unit; *W*_*f*_ is weight; *b*_*t*_ is the bias; σ is the sigmoid. Input gate and output gate can be expressed by Eqs. (11)–(14):

11 $$ i_{t} = \sigma \left( {W_{i} *\left[ {x_{t} ,h_{t - 1} ,C_{t - 1} } \right] + b_{i} } \right) $$

12 $$ C_{t} = f_{t} + i_{t} *\tanh \left( {W_{i} *\left[ {x_{t} ,h_{t - 1} ,C_{t - 1} } \right]} \right) + b_{i} $$

13 $$ o_{t} = \sigma \left( {W_{o} *\left[ {x_{t} ,h_{t - 1} ,C_{t} } \right] + b_{o} } \right) $$

14 $$ h_{t} = \tanh \left( {C_{t} } \right)*o_{t} $$

In the formula, *C*_*t−1*_ is the output status of the input gate; *h*_*t*_ is the output of the LSTM unit. The LSTM is fed by the softmax output of the CNN as the feature.

**Figure 4:**
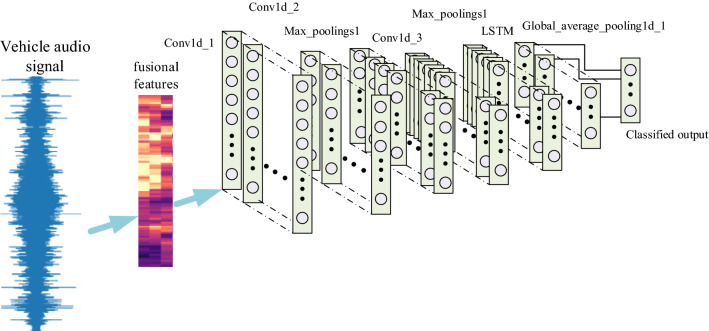
Structure of the hybrid neural network classifier.

The hybrid neural network classifier proposed in this paper is shown in Fig. 4. It includes 1 input layer, 3 convolution layers, 2 Batch Normalization layers, 2 pooling layers. Conv1d represents 1-dimensional convolution and LSTM adopts one time step.

## Experimental results

### Effectiveness of novel feature

To verify the validity of the novel feature which proposed in this paper with support vector machine (SVM) and hybrid neural network classifiers (HNN), we use the data of an extensive real world experiment (http://www.ecs.umass.edu/~mduarte/Software.html), which includes vehicle sensor signals for both assault amphibian vehicle and Dragon Wagon. This paper adopts 180 vehicle signal data collected from the same sampling location. In this experiment, we divided the dataset into training set and test set. Due to the small amount of experimental data, and in order to utilize as much as possible the limited number of datasets, we used a tenfold cross-validation method to Evaluate all methods where 90% of the data was selected for training, 10% for testing and for 10 non-overlap test dataset repeat 10 times. It makes all samples available for training the model. Firstly, we preprocessed these data, the process is as follows: the length of the frame and window are both 128, the frame shifting is 64, resampling to 44.1 kHz. We spliced two of the three features and compared with the novel features proposed in this paper in order to verify the effectiveness of the novel feature proposed in this paper. To avoid the contingency of the experiment, we use multiple experiments and take the average as the experimental result.

**Figure 5:**
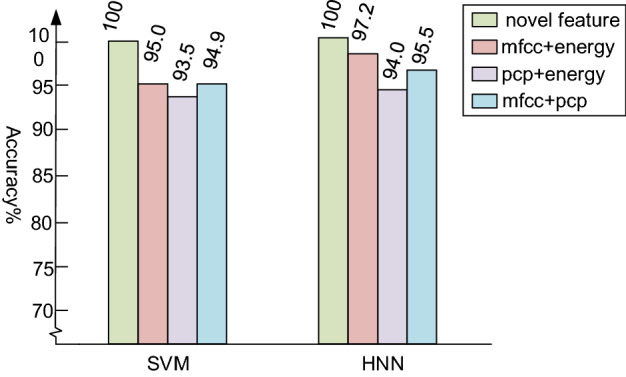
The accuracy of different feature experiment.

The experimental results are shown in Fig. 5. As we can see from the result that the novel feature can capture audio information from different aspects, retain information from the psychoacoustic feature, the acoustic feature and the perceptual feature, which can represent the vehicle audio information more comprehensively and make the recognition more accurately. It can also be seen from the figure that compared with other features, the method combining these three feature vectors can improve the recognition accuracy by nearly 7%. In the meantime, the novel feature is unidimensional, so the complexity of the algorithm has not increased. The feature containing MFCC has a better recognition effect, since it is a good simulation of human auditory sensation. As shown in Fig. 5, when using the tenfold crossover method, the average accuracy of the novel features as input features can reach 100%, which can fully prove the superiority of the novel features extracted in this paper, and the recognition rate is higher than other features. On the other hand, in different numbers of test sets, the recognition accuracy with HNN which proposed in this paper is higher than SVM.

### Verification experiment by augmenting dataset

**Figure 6:**
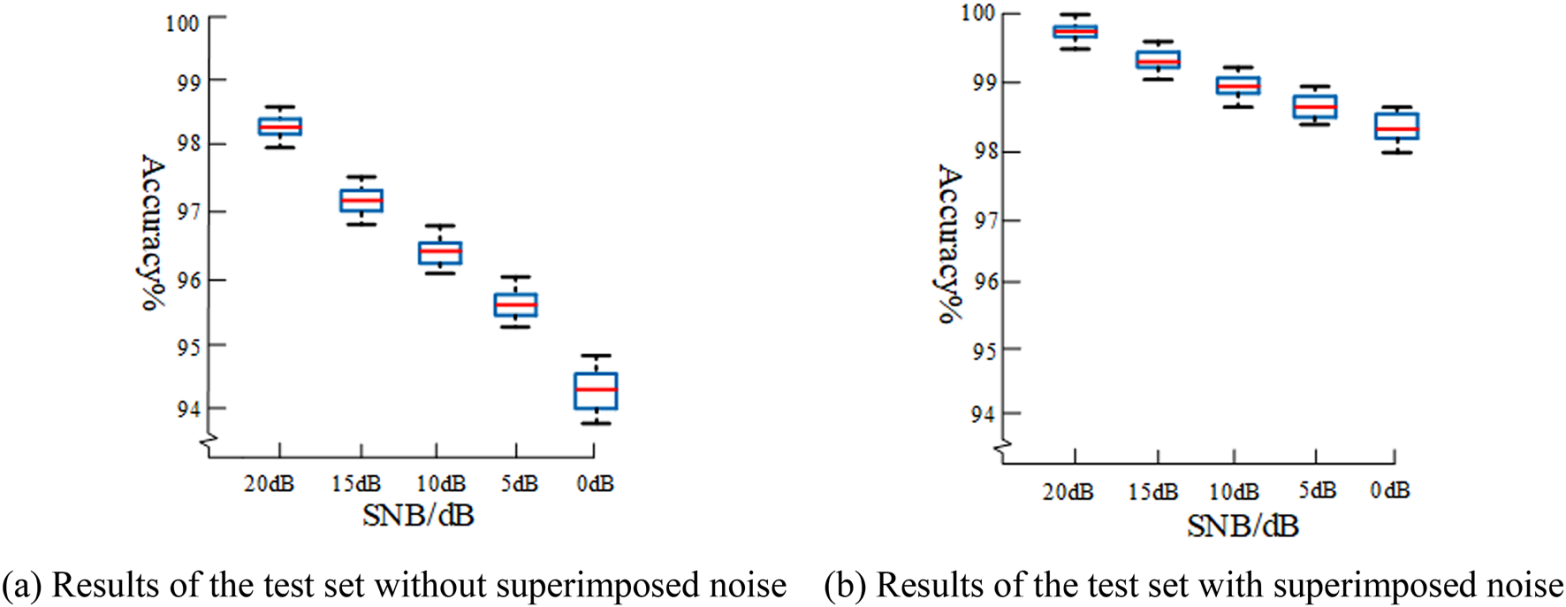
Comparative experiments.

In order to verify the generalization ability of the vehicle recognition system, the city noise is superimposed on the vehicle audio signal, and the signal noise ratio (SNR) is 20 dB, 15 dB, 10 dB, 5 dB and 0 dB respectively. We randomly select 80% of the data sets to add noise with different signal-to-noise ratios, so that on the one hand, the size of data sets can be expanded, and on the other hand, each vehicle can be simulated in different environments. After training, we tested the recognition accuracy of the model using the test set. Here, the test set can be divided into two parts, one part is the audio with noise added, and the other part is the original data set without noise. We use the same data as previous experiment under the same conditions. We select the novel feature as the input vector. We also use the tenfold cross-validation method described in “Effectiveness of novel feature” to divide the data set. Dropout rate is set to 0.25. The initial learning rate is set to 0.001. Moving average attenuation coefficient is set to 0.9. Stochastic gradient descent algorithm (SGD) is used to update the learning rate. In order to reflect the performance of dataset augmentation more visually, we trained the original signal without superimposing noise under the same conditions and tested with the noisy signal. We performed five experiments for each SNR signal to guarantee the reliability. The training set accounted for 70% of the total data set. The experimental recognition accuracy is shown in Fig. 6.

As shown in Fig. 6., when experiment with dataset augmentation and the SNR is 0 dB, the recognition accuracy is 98.46%. However, the accuracy of the experiment without dataset augmentation is 94.5%. When the signal to noise ratio is in the range of 0 dB to 20 dB, the stability and accuracy of the model with dataset augmentation are significantly higher than the latter. The anti-interference ability of the model is greatly improved. Furthermore, it is verified that this recognition system has high accuracy in different SNR.

### Verification experiment of sensor acquisition data

**Figure 7:**
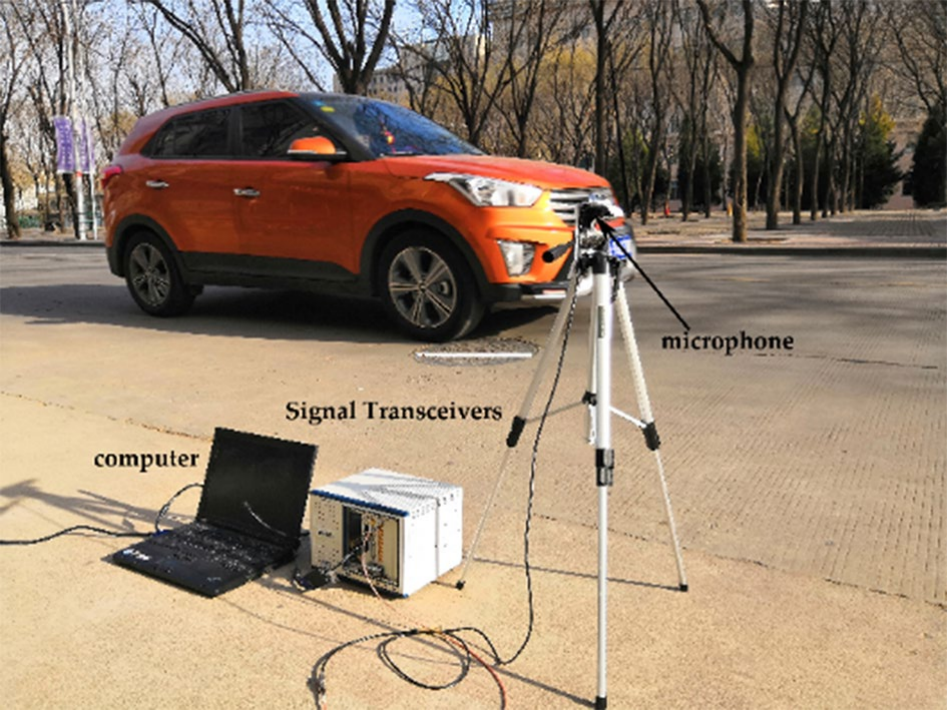
The process of audio recording.

**Figure 8:**
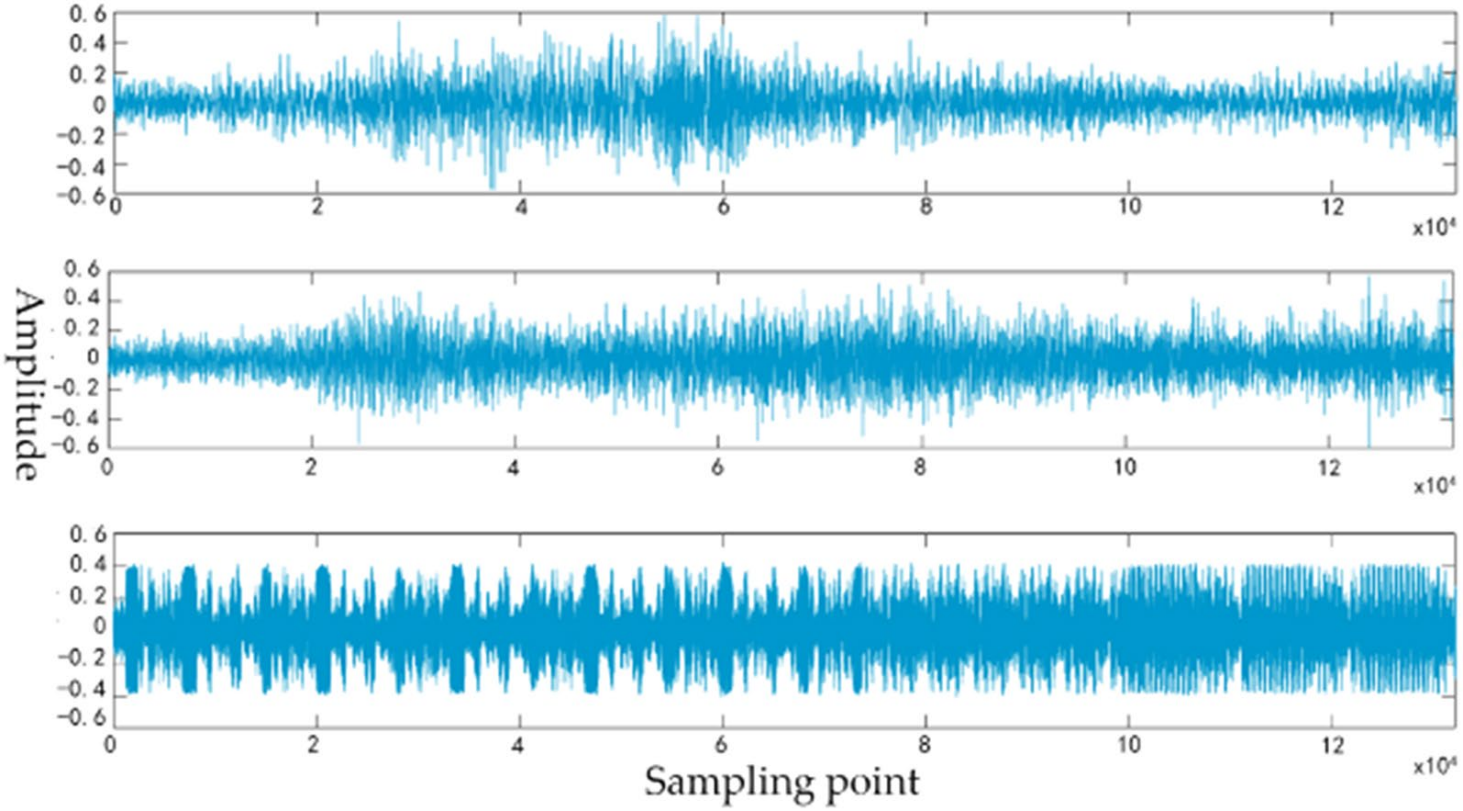
The vehicle audio waveform.

This experiment aims to verify the robust of the recognition system, we recorded 10 different kinds of vehicle audio, including YUTONG bus, JAC truck, HAVAL H5, SGMW, RANGE ROVER, JETTA, HYUNDAI IX35, BYD e6, JAC V7, and train. The recording process of the audio is shown in Fig. 7. The collected equipment includes a microphone, a PXI Vector Signal Transceivers and a computer. The number of each vehicle sample is 100. The sampling frequency is 44.1 kHz. The audio contains the vehicle’s engine sound, horn sound, brake sound and the tire friction sound. Due to the interference noise from different vehicles when collecting, we intercepted 132,300 sample points containing the largest ingredient of the main vehicles as the sample point data. The collected vehicle audio waveform is shown in Fig. 8. To verify the impact of the LSTM layer on the recognition accuracy of the system, under the same experimental condition, we randomly selected 80% of the training set to superimpose noise.

We experimented with LSTM layer and without LSTM layer separately and performed 5 experiments according to the test set ratio of 20%, 25%, 30%, 35%, 40% and 45% respectively. The accuracy and recall of experiments are shown in Table 1.

**Table 1:** The accuracy and recall of the experiment.

Test set (%)	LSTM layer		No LSTM layer	
	Accuracy (%)	Recall (%)	Accuracy (%)	Recall (%)
20	98.80	98.23	96.05	96.00
25	98.10	98.06	95.00	95.02
30	97.86	97.50	94.73	94.95
35	97.20	96.97	94.20	94.00
40	97.00	96.56	93.00	92.76
45	96.96	96.28	92.56	92.32
Average	97.65	97.27	94.26	94.18

As shown in Table 1, adding LSTM layer to the networks can improve the recognition rate of 3.39%. The reason for this effect is that the LSTM unit has a certain memory for the front and back frame information of the audio, so the correlation between the frictional sound in the signal and the engine sound appears obviously. The LSTM unit has great advantages in modelling time series. Therefore, adding the LSTM layer to the neural network classifier has a great effect on improving the system performance.

**Figure 9:**
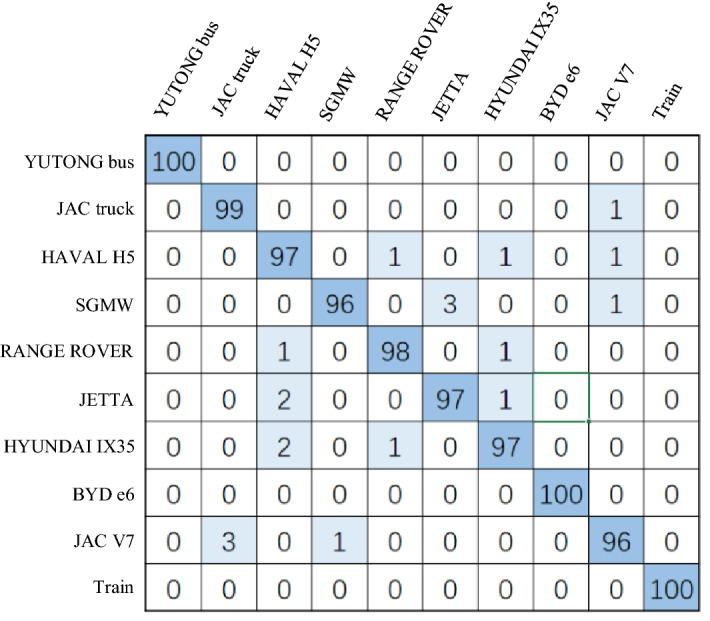
The confusion matrix of the experiment.

**Figure 10:**
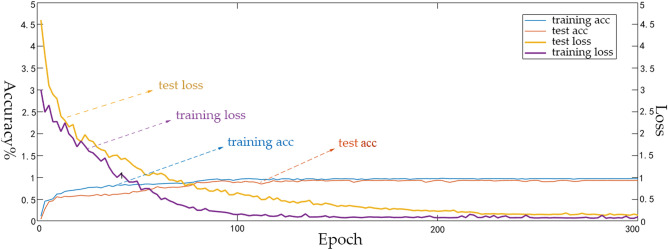
The accuracy and loss of the experiment.

Figure 9 is the confusion matrix of the experiment. We selected 700 of them as training sets. It can be seen that the recognition rate of large vehicles is very high, the reasons are that the large vehicles are heavy and the friction between tires and ground is more characteristic than the other small vehicles. Furthermore, large vehicles use diesel engines, the distinctive noise is caused largely by the way the fuel ignites. BYD e6 is the new energy vehicle, its motor drive will not have the same vibration and noise as the internal combustion engine, so the characteristics are relatively obvious compared with gasoline and diesel engines, so it has a good recognition effect. Figure 10 shows the accuracy and loss function of the experiment. We can see that the recognition accuracy tends to be stable after 150 iterations, and has remained at around 98%. The loss function tends to be stable about 200 iterations, and the test set loss function curve has a high similarity with the training set, tending to 0.05, indicating that the model has strong robustness.

**Figure 11:**
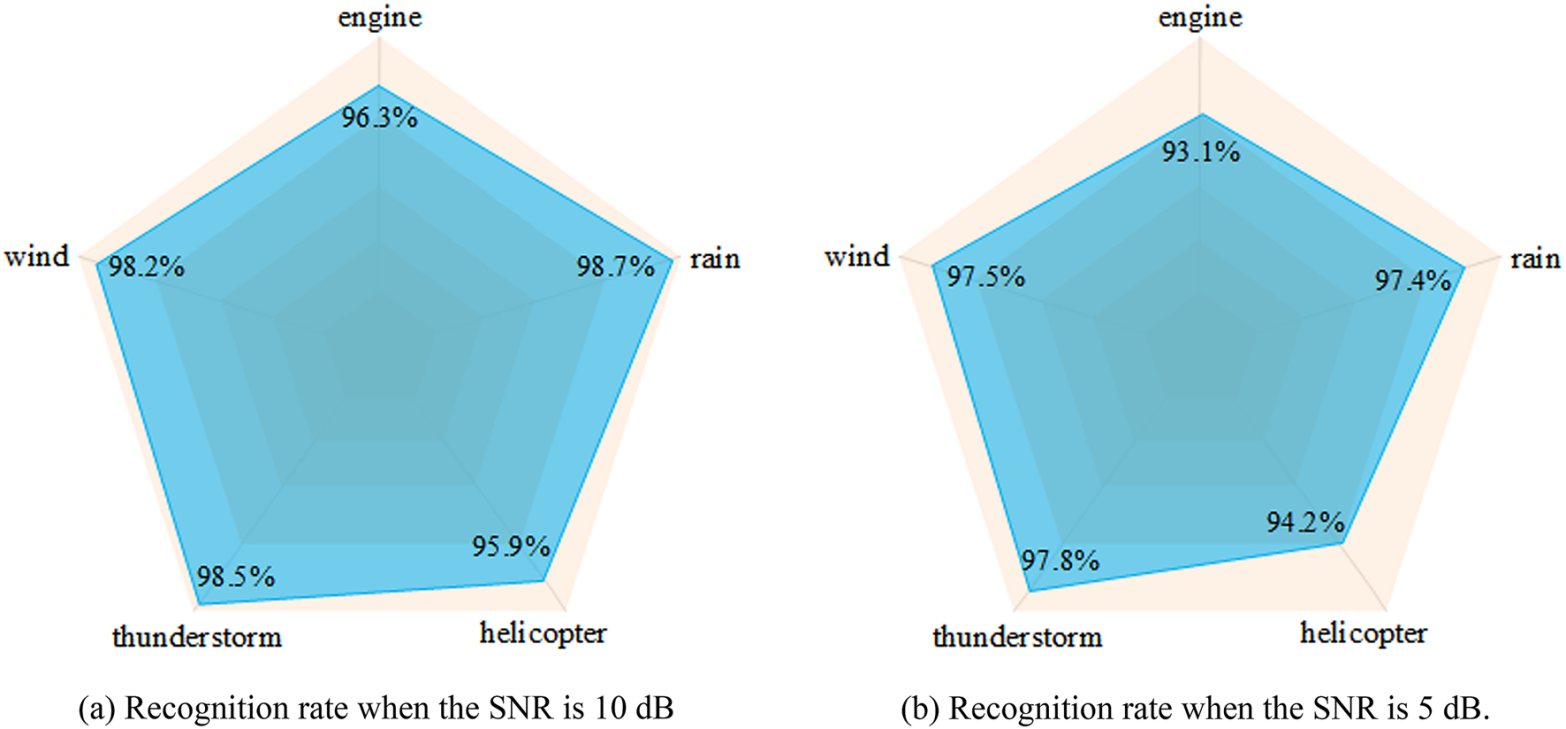
The accuracy and loss of experiment.

To verify the impact of different types of noise, we randomly selected 80% of the audio superimposing the wind noise, engine noise, rain noise, thunderstorm noise and helicopter noise which contain in the ESC-50 data set on the vehicle audio with the SNR of 10 dB and 5 dB respectively. After extracting features, we put them into the hybrid neural network to train and performed 5 time experiments every kind of noise, then took the average. The comparison experimental are shown in Fig. 11.

As shown in Fig. 11, the recognition rate can be stabilized at around 97.5%, when superimposing the wind, rain and thunderstorm noise; But when we superimposed engine and helicopter noise, it has a certain impact on the recognition rate of the system, the reason is that both types of noise have mechanical vibration and is similar to the vehicle audio. Therefore, when we extract features, this mechanical vibration is easily extracted as the vehicle audio characteristics, which affecting the accuracy of recognition. The recognition accuracy will remain at 93%, even if there is some impact. It can be proved that under the complex environmental noise, the recognition rate of the system can be stabilized above 93%.

## Conclusion

In the vehicle identification system, the quality of extracting feature and the performance of the classifier affect the accuracy of recognition. In this paper, we spliced the Mel frequency cepstrum coefficient, the reformative pitch class profile and the short-term energy as the input vector of the hybrid neural networks. The Mel frequency cepstrum coefficient can reflect the auditory properties of human ear. The reformative pitch class profile can reflect the tone of the vehicle audio. When the background noise is low, the short-term energy can reflect the audio characteristics very well. Compared with other identification system for identifying the vehicle type, the developed system in this paper has many advantages. Compared with the support vector machine, for the extraction of different features of audio, in general, the hybrid neural network classifier has a higher recognition effect. Compared with the other features, the novel features can reflect the characteristic of vehicle audio very well, the recognition rate can reach 100% on the data of an extensive real word experiment. The recognition system we proposed can make recognition rate to 98% when the vehicle audio is collected by the microphone. When we destroy the training set randomly, the accuracy of this system is higher, so destroying the training set randomly can greatly improve the anti-noise ability. We proposed the hybrid neural network combining the convolutional neural network with the LSTM unit, which has better fit to time series of vehicle audio and can improve the vehicle identification accuracy significantly. Experimental results that the LSTM unit can improve the recognition accuracy by 3.39%.

In future, we would like to collect more vehicle audio and perform the experiment with the new dataset to confirm if the identification system has more advantages than other system. This paper only superimposes different types of noise on the actual collected vehicle audio. In order to better verify the feasibility of the algorithm in this paper, future work should collect vehicle sounds in real environments, such as rainy weather and strong winds.

## References

[1] KrishnaswamyRangarajan A, Purushothaman R (2020). Disease classification in eggplant using pre-trained VGG16 and MSVM. J. Sci. Rep..

[2] Lee B, Cho K-H (2016). Brain-inspired speech segmentation for automatic speech recognition using the speech envelope as a temporal reference. J. Sci. Rep..

[3] Thompson J (2019). Relevant word order vectorization for improved natural language processing in electronic health records. J. Sci. Rep..

[4] Chang, B. Performance evaluation and prediction of rudders based on machine learning technology. *J. Proc. Inst. Mech. Eng. Part G J. Aerosp. Eng.* **233**. 5746–5757 (2019).

[5] Jin M, Deng W (2018). Predication of different stages of Alzheimer's disease using neighborhood component analysis and ensemble decision tree. J. Neurosci. Methods..

[6] Parmley KA, Higgins RH, Ganapathysubramanian B, Sarkar S, Singh AK (2019). Machine learning approach for prescriptive plant breeding. J. Sci. Rep..

[7] Razaghi-Moghadam Z, Nikoloski Z (2020). Supervised learning of gene regulatory networks. J. Curr. Protoc. Plant Biol..

[8] Pagan DC, Phan TQ, Weaver JS, Benson AR, Beaudoin AJ (2019). 4 Unsupervised learning of dislocation motion. J. Acta Mater..

[9] Engelen, J.E.v. & Hoos, H.H. A survey on semi-supervised learning. *J. Mach. Learn.* **109**, 373–440 (2019).

[10] Sheth A, Gaur M, Kursuncu U, Wickramarachchi R (2019). Shades of knowledge-infused learning for enhancing deep learning. J. IEEE Internet Comput..

[11] Wang, Z., Zhang, X., Yang, X. & Xia, W. Robust vehicle detection on multi- resolution aerial images. *J. IOP Conf. Ser. Mater. Sci. Eng*. **719**, 012064 (2020).

[12] Hassaballah M, Kenk MA, El-Henawy IM (2020). Local binary pattern-based on-road vehicle detection in urban traffic scene. Pattern Anal. Appl..

[13] Gong-ping, Y., Yi-ping, T., Wang-ming, H. & Qi, C. Vehicle category recognition based on deep convolutional neural network. *J. Zhejiang Univ. (Eng. Sci.).* **52**, 694–702 (2018).

[14] Inagaki K, Sato S, Umezaki T (2003). A recurrent neural network approach to rear vehicle detection which considered state dependency. J. Syst. Cybern. Inform..

[15] Wang, T. & Zhu, Z. Multimodal and multi-task audio-visual vehicle detection and classification. *J. *In *2012 IEEE Ninth International Conference on Advanced Video and Signal-Based Surveillance (AVSS),* Vol. 47, 440–446 (2012).

[16] Chen N, Downie JS, Zhu Y, Xiao H-D (2015). Cochlear pitch class profile for cover song identification. J. Appl. Acoust..

[17] Krishnaswamy Rangarajan A, Purushothaman R (2020). Disease classification in eggplant using pre-trained VGG16 and MSVM. Sci. Rep..

